# *Pseudocedrela kotschyi*: a review of ethnomedicinal uses, pharmacology and phytochemistry

**DOI:** 10.1080/13880209.2021.1950776

**Published:** 2021-07-20

**Authors:** Alhassan M. Alhassan, Qamar Uddin Ahmed, Ibrahim Malami, Zainul Amiruddin Zakaria

**Affiliations:** a Department of Pharmaceutical and Medicinal Chemistry, Faculty of Pharmaceutical Sciences, Usmanu Danfodiyo University, Sokoto, Nigeria; b Pharmacognosy Research Group, Department of Pharmaceutical Chemistry, Kulliyyah of Pharmacy, International Islamic University Malaysia, Kuantan, Malaysia; c Department of Pharmacognosy and Ethnopharmacy, Faculty of Pharmaceutical Sciences, Usmanu Danfodiyo University, Sokoto, Nigeria; d Department of Biomedical Science, Faculty of Medicine and Health Sciences, Universiti Putra Malaysia, Serdang, Malaysia; e Laboratory of Halal Science Research, Halal Products Research Institute, Universiti Putra Malaysia, Serdang, Malaysia

**Keywords:** Traditional uses, scientific claims, bioactive compounds, limonoid orthoacetates, kotschyins, kostchyienones, pseudrelones, toxicity

## Abstract

**Context:**

*Pseudocedrela kotschyi* (Schweinf) Harms (Meliaceae) is an important medicinal plant found in tropical and subtropical countries of Africa. Traditionally, *P. kotschyi* is used in the treatment of various diseases including diabetes, malaria, abdominal pain and diarrhoea.

**Objective:**

To provide an overview of traditional medicinal claims, pharmacological properties, and phytochemical principles of *P. kotschyi* as a basis for its clinical applications and further research and development of new drugs.

**Methods:**

Through interpreting already published scientific manuscripts retrieved from different scientific search engines, namely, Medline, PubMed, EMBASE, Science Direct and Google scholar databases, an up-to-date review on the medicinal potentials of *P. kotschyi* from inception until September, 2020 was compiled. ‘*Pseudocedrela kotschyi*’, ‘traditional uses’, ‘pharmacological properties’ and ‘chemical constituents’ were used as search words.

**Results:**

At present, more than 30 chemical constituents have been isolated and identified from the root and stem bark of *P. kotschyi*, among which limonoids and triterpenes are the main active constituents. Based on prior research, *P. kotschyi* has been reported to possess anti-inflammatory, analgesic, antipyretic, anthelminthic, antimalaria, anti-leishmaniasis, anti-trypanosomiasis, hepatoprotective, antioxidant, antidiabetic, antidiarrheal, antimicrobial, and anticancer effects.

**Conclusions:**

*P. kotschyi* is reported to be effective in treating a variety of diseases. Current phytochemical and pharmacological studies mainly focus on antimalaria, anti-leishmaniasis, anti-trypanosomiasis and anticancer potential of the root and stem bark of *P. kotschyi*. Although experimental data support the beneficial medicinal properties of this plant, there is still a paucity of information on its toxicity profile. Nonetheless, this review provides the basis for future research work.

## Introduction

Traditional medicinal plants have been an essential source of remedy for various illnesses since ancient times. Preparations of plant materials such as infusion, decoction, powder, or paste have been used in various traditional practices in different parts of the world. People living in Africa and Asia make use of herbal medications to supplement the conventional medicine practice (Ekor 2014). There has been an increasing interest in the usage of herbal medicines in recent years. About 80% of the world’s population is using phytotherapeutic medicines (Balekundri and Mannur 2020). The WHO estimated that the size of the global market for herbal products was USD 60 billion in the year 2000 and this is expected to grow 7% per annum towards USD 5 trillion by the year 2050 (Tan et al. 2020). Several analyses have clearly verified traditional claims of numerous medicinal plants leading to the commercialisation of the many herbal products and their nomination as leads within the development of pharmaceutical medication (Williams 2021). Many clinically useful drugs have been discovered based on the knowledge derived from the ethnomedicinal applications of various herbal materials (Balunas and Kinghorn 2005).

*Pseudocedrela kotschyi* (Schweinf.) Harms (Meliaceae) is an important medicinal plant found in the tropical and subtropical countries of Africa. This plant has been extensively used in the African traditional medicine system for the treatment of a variety of diseases, particularly as analgesic, antimicrobial, antimalarial, anthelminthic, and antidiarrheal agents. The main focus of this review was to establish the ethnopharmacological uses and medicinal characteristics of *P. kotschyi* and highlight its potential as future drug for the treatment of various tropical diseases.

## Methodology

Scientific manuscripts on *P. kotschyi* were retrieved from different scientific search engines. Literature search was carried out on PUBMED using *Pseudocedrela kotschyi* as key words. Additional literature searches on Medline, EMBASE, Science Direct and Google scholar databases were done using pharmacological activity, chemical constituents and traditional uses of *Pseudocedrela kotschyi* as search terms. Literature published on the topic in English language from inception until September, 2020 were collected, analysed and an up-to-date review on the medicinal potential of *P. kotschyi* was compiled.

### Geographical distribution

*P. kotschyi* (common name: dry zone cedar) is a medicinal plant. Other common names of *P. kotschyi* are Tuna (Hausa) and Emi gbegi in Yoruba. It is found in tropical and subtropical countries of Africa which include Nigeria, Cote d’Ivoire, Senegal, Ghana, Democratic Republic of Congo, and Uganda. The plant often grows as a medium sized tree of about 12–20 ft high (Ayo et al. 2010; Alain et al. 2014; Alhassan et al. 2014). Below is the taxonomical classification of *P. kotschyi* (Hassler 2019).

***Classification***

Kingdom  Plantae

Phylum  Tracheophyta

Class   Magnoliopsida

Order    Sapindales

Family   Meliaceae

Genus    *Pseudocedrela*

Species   *kotschyi*

### Ethnomedicinal uses

Different parts of *P. kotschyi* are used in the traditional treatment of various diseases. The root is used in the treatment of leprosy (Pedersen et al. 2009), epilepsy, dementia (Kantati et al. 2016), diabetes (Salihu Shinkafi et al. 2015), malaria, abdominal pain, diarrhoea (Ahua et al. 2007), toothache and gingivitis (Tapsoba and Deschamps 2006). The root is also used as a chewing stick for tooth cleaning and enhancement of oral health (Wolinsky and Sote 1984; Olabanji et al. 2007; Adeniyi et al. 2010). The leaf is used in the treatment of female infertility (Olabanji et al. 2007), intestinal worms (Koné et al. 2005) and malaria (Asase et al. 2005). The stem bark is used in the treatment of cancer (Saidu et al. 2015), infantile dermatitis (Erinoso et al. 2016), stomach ache (Asase et al. 2005), toothache (Kayode and Sanni 2016), high blood-pressure, skin diseases, and haemorrhoids (Nadembega et al. 2011).

### Phytochemistry

Phytochemical investigations revealed that *P. kotschyi* contains a variety of pharmacological active secondary metabolites. A total of 32 compounds have so far reported to have been isolated from the plant which mainly include limonoids, triterpenes, and flavonoids.

Limonoids are modified triterpenes which are highly oxygenated and have a typical furanylsteroid as their core structure (Roy and Saraf 2006). They are also known as tetraterpenoids. Limonoids are rare natural products which occur mainly in plants of Meliaceae and Rutaceae families and less frequently in the Cneoraceae family (Tan and Luo 2011).

**Figure 1.**
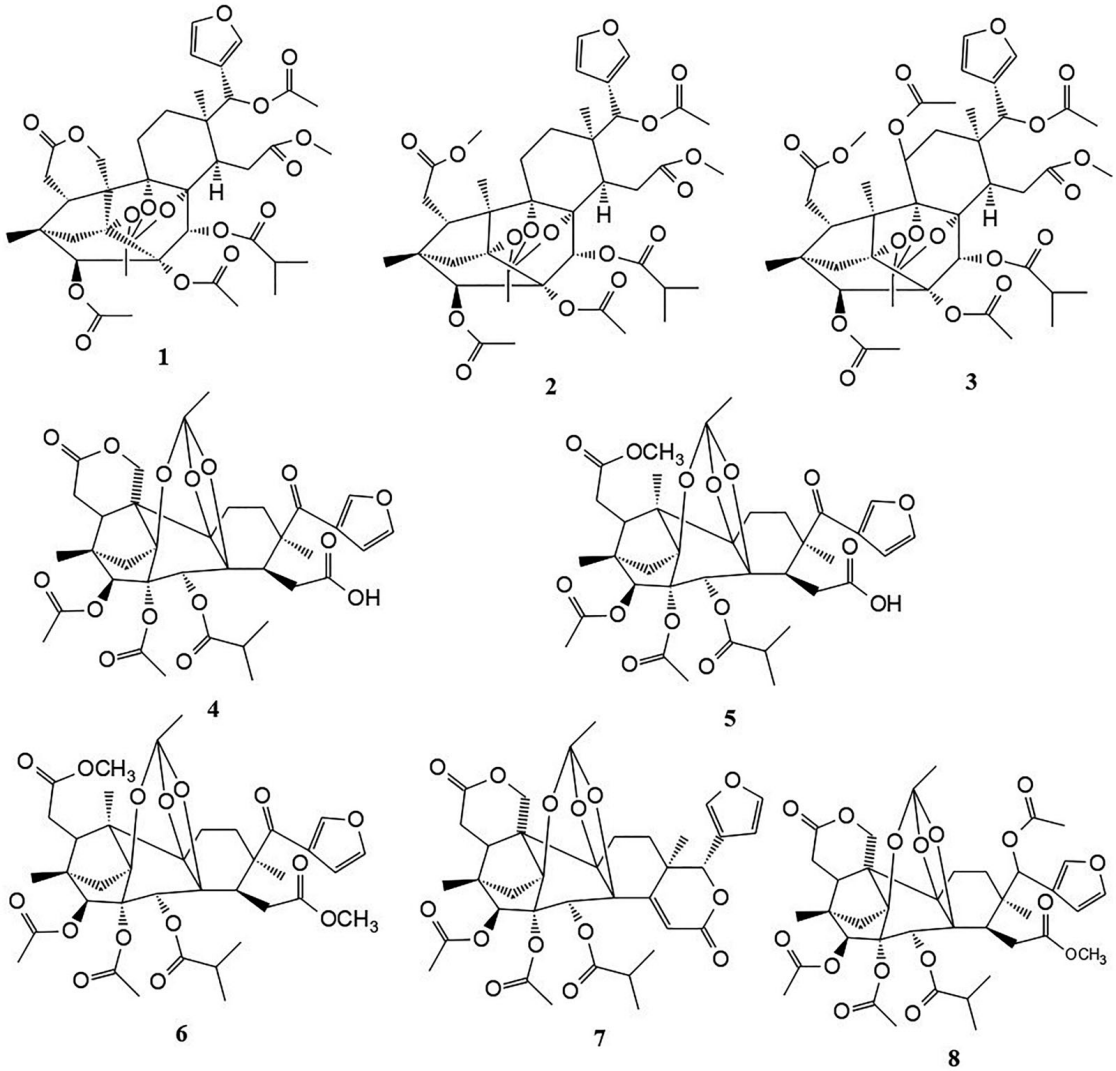
Phragmalin-type limonoid orthoacetates isolated from the roots of *P. kotschyi*.

Several phragmalin-type limonoid orthoacetates (Figure 1) have reportedly been isolated from the of roots of this plant, namely, kotschyins A**–**H (**1–8**) (Hay et al. 2007; Dal Piaz et al. 2012). These compounds are complex with a very high degree of oxidation and rearrangement as compared to the parent limonoid structure.

**Figure 2.**
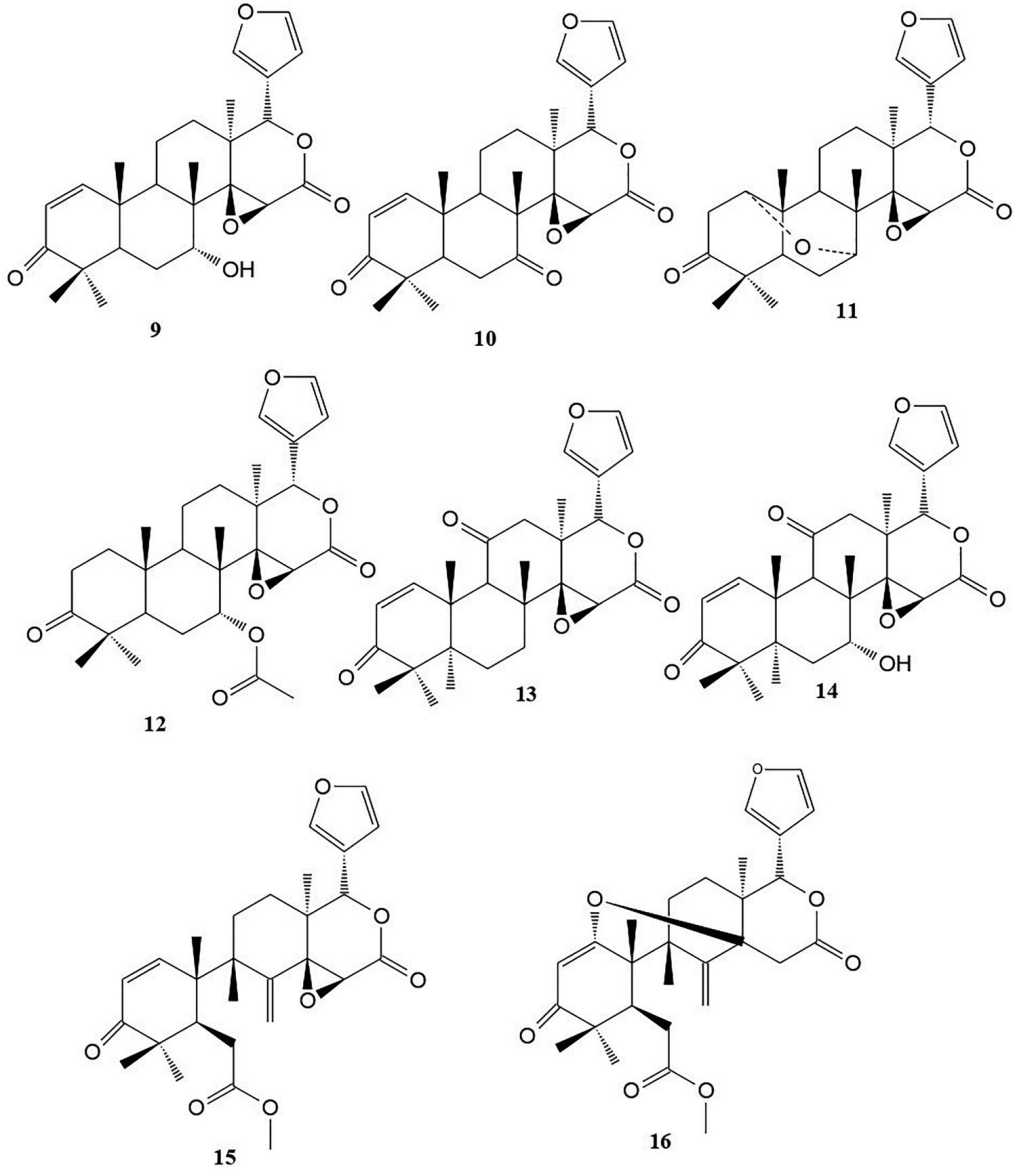
Limonoid derivatives isolated from the roots of *P. kotschyi*.

**Figure 3.**
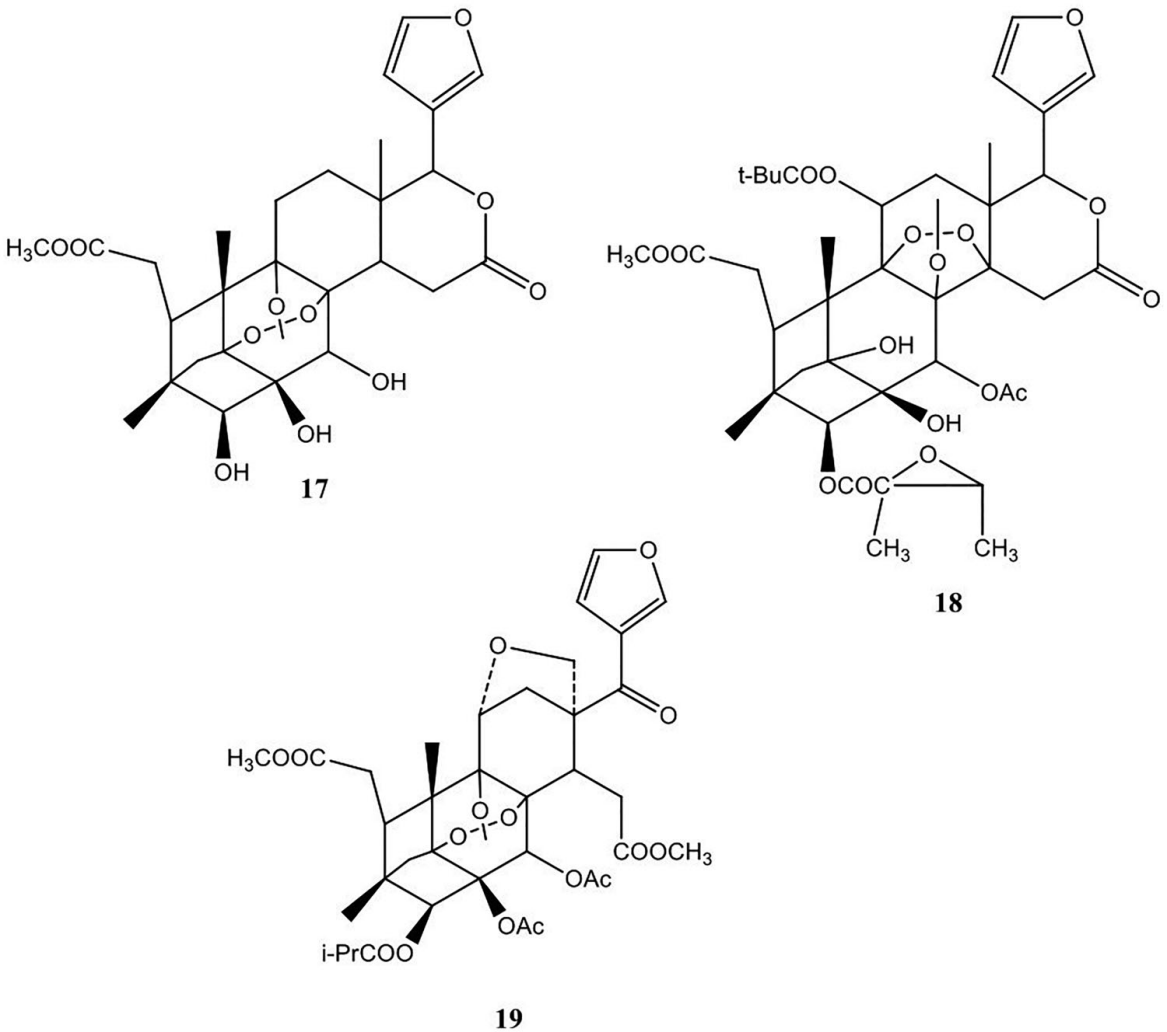
Limonoid derivatives isolated from the bark of *P. kotschyi*.

Other limonoid derivatives (Figure 2) found in the roots and stem bark of *P. kotschyi* are 7-deacetylgedunin **(9)**, 7-deacetyl-7-oxogedunin **(10)** (Hay et al. 2007), 1α,7α epoxy-gedunin (**11**), gedunin (**12**) (Dal Piaz et al. 2012), kostchyienones A (**13**) and B (**14**), andirobin (**15**) methylangolensate (**16)** (Sidjui et al. 2018). Additional limonoids derivatives (Figure 3) that were isolated from the *P. kotschyi* bark include pseudrelones A**–**C (**17–19**) (Taylor 1979). The pseudrelones also have a phragmalin nucleus with orthoacetate function but they have a lesser degree of oxidation than the kotschyins.

**Figure 4.**
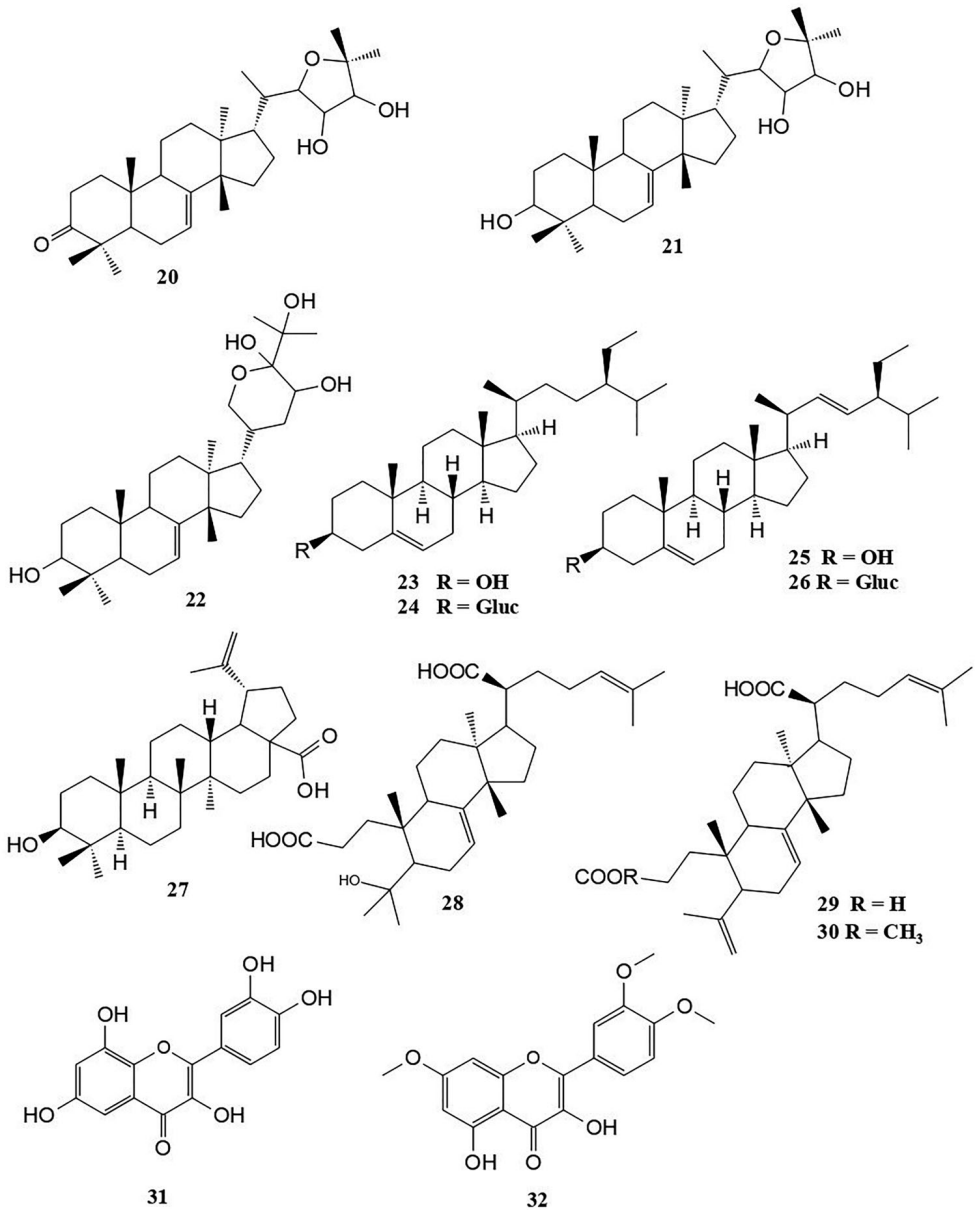
Triterpenes and flavonoids isolated from the roots of *P. kotschyi*.

The steroids isolated from this plant (Figure 4) include odoratone (**20**), spicatin (**21**), 11-acetil-odoratol (**22**) (Dal Piaz et al. 2012), β-sitosterol (**23**), 3-*O*-β-d-glucopyranosyl β-sitosterol (**24**) stigmasterol (**25**), 3-*O*-β-d-glucopyranosyl stigmasterol (**26**) betulinic acid (**27**) (Sidjui et al. 2018). Three secotirucallane triterpenes were also isolated from the stem bark of *P. kotschyi*. These include, 4-hydroxy-3,4-secotirucalla-7,24-dien-3,21-dioic acid (**28**), 3,4-secotirucalla-4(29),7,24-trien-3,21-dioic acid (**29**) and 3-methyl ester 3,4-secotirucalla-4(28),7,24-trien-3,21-dioic (**30**) (Figure 4) (Mambou et al. 2018). Two flavonoids, namely, 3,6,8-trihydroxy-2-(3,4-dihydroxylphenyl)-4H-chrom-4-one (**31**) and quercetin, 3,4′,7-trimethyl ether (**32**) (Figure 4) have also been isolated from the roots of this plant (Sidjui et al. 2018).

The GCMS analysis of essential oils from root and stem of *P. kotschyi* indicated that both oils contain mainly sesquiterpenoids. These include, α-cubebene, α-copaene, β-elemene, β-caryophyllene, *trans*-α-bergamotene, aromadendrene, (*E*)-β-farnesene, α-humulene, allo-aromadendrene, γ-muurolene, farnesene, germacrene D, β-selinene, α-selinene, α-muurolene, γ-cadinene, calamenene, δ-cadinene, cadina-1,4-diene, α-calacorene, α-cadinene, β-calacorene, germacrene B, cadalene, *epi*-cubebol, cubebol, spathulenol, globulol, humulene oxide II, *epi*-α-cadinol, *epi*-α-muurolol, α-muurolol, selin-11-en-4-α-ol, α-cadinol and juniper camphor. The stem bark oil was found to comprise largely of sesquiterpene hydrocarbons (79.6%), with δ-cadinene (31.3%) as the major constituents. While the oxygenated sesquiterpenes were found to be abundant in the root with cubebols (32.5%) and cadinols (17.9%) as the major constituents (Boyom et al. 2004).

### Pharmacological activity

The ethnomedicinal claims for the efficacy of *P. kotschyi* in the treatment of various diseases have been confirmed by numerous relevant scientific studies. Several pharmacological investigations have been carried out to confirm the traditional medicinal uses of the roots, stem bark, and leaves of *P. kotschyi*. A wide range of pharmacological activities such as analgesic, antipyretic, anthelminthic, antimalaria, anti-leishmaniasis, hepatoprotective, antioxidant, and antimicrobial, have been reported by researchers so far.

### Anti-inflammatory, analgesic and antipyretic activities

Inflammation is an adaptive response that is triggered by noxious stimuli or conditions, such as tissue injury and infection (Medzhitov 2008; Ahmed 2011). Inflammatory response involves the secretion of several chemical mediators and signalling molecules such as nitric oxide (NO), and proinflammatory cytokines, including tumour necrosis, factor-α (TNF-α), interferon-γ (IFNγ), lipopolysaccharides (LPS) and interleukins (Medzhitov 2008). Even though inflammatory response is meant to be a beneficial process of restoring homeostasis, it is often associated with some disorders like pain and pyrexia due to the secretion of the chemical mediators (Bielefeldt et al. 2009; Garami et al. 2018). Chronic secretion of proinflammatory cytokines is also associated with development of diseases such as cancer and diabetes. Hence, anti-inflammatory agents represent an important class of medicines. Extracts and phytoconstituents of *P. kotschyi* have been reported to possess anti-inflammatory, analgesic and antipyretic properties.

The administration of methanol crude extract of *P. kotschyi* stem bark at a dose of 200 mg/kg/day and its butanol and chloroform fractions have been shown to produce significant analgesic activity when evaluated with a mice model. The extracts and fractions decreased the number of writhes by 88–92% during the acetic acid induced writhing assay (Abubakar et al. 2016). Akuodor et al. (2013) investigated the antipyretic activity of ethanol extract of *P. kotschyi* leaves on yeast and amphetamine induced hyperpyrexia in rats. They reported that the leaf extract (50, 100 and 150 mg/kg i.p.) displayed a significant (*p* < 0.05) dose-dependent decrease in pyrexia.

Scientific investigations have shown that 7-deacetylgedunin (**9)** had significant anti-inflammatory activity. Compound **9** was reported to significantly inhibit lipopolysaccharide induced nitric oxide in murine macrophage RAW 264.7 cells with an IC_50_ of 4.9 ± 0.1 μM. It also produced the downregulation of mRNA and protein expression of inducible nitric oxide synthase (iNOS) at a dose of 10 µM (Sarigaputi et al. 2015). These findings suggest that compound **9** produces its anti-inflammatory effect through the modulation of NO production. Chen et al. (2017) investigated the anti-inflammatory activity of compound **9** in C57BL/6 mice. Their result showed that its intraperitoneal administration at a dose of 5 mg/kg body weight for two consecutive days significantly decreased LPS-induced mice mortality by 40%. The above findings demonstrate that compound **9** is a promising anti-inflammatory agent from this plant. The anti-inflammatory effect of this compound perhaps accounts for the analgesic and antipyretic properties of the *P. kotschyi* extracts.

### Antiparasitic activity

Parasitic diseases are among the foremost health problems today, especially in tropical countries of Africa and Asia. Diseases such as malaria, leishmaniasis, trypanosomiasis and helminthiasis affect millions of people each year causing high morbidity and mortality, particularly, in developing countries (Hotez and Kamath 2009). Hence, there is an urgent need for new drugs to treat and control of these diseases.

Extracts obtained from different parts of *P. kotschyi* have been reported to possess activity against several human parasites. Ahua et al. (2007) investigated the anti-leishmaniasis activity of *P. kotschyi* including several other plants against *Leishmania major*. The dichloromethane extract of *P. kotschyi* roots (at a dose of 75 µg/mL) exhibited a marked activity (>90% mortality) against the intracellular form of the parasite which is pathogenically significant for humans.

In another study, the anthelminthic activity of an ethanol extract of *P. kotschyi* roots against *Haemonchus contortus* (a pathogenic nematode found in small ruminants) was evaluated. The researchers discovered that the ethanol extract possessed larvicidal activity against the helminth with a LC_100_ of 0.02 µg/mL (Koné et al. 2005). The aqueous stem bark extract of this plant (50 mg/mL) has also been demonstrated to exert anthelminthic activity against *Lumbricus terrestris* with 25.4 min death time (Ukwubile et al. 2017).

The antimalarial effect of *P. kotschyi* on malaria parasite has been reported in several research manuscripts. Christian et al. (2015) investigated the suppressive and curative effect of ethanol extract of *P. kotschyi* leaves against malaria in *Plasmodium berghei berghei* infected mice. The results obtained showed that oral administration of the extract (100–400 mg/kg/day) exhibited a significant antimalarial effect which is evident by the suppression of parasitemia and prolong life of infected animals. In an another study, methanol extract of the *P. kotschyi* leaves at an oral dose of 200 mg/kg/day was found to reduce parasitemia by 90.70% in *P. berghei berghei* infected mice after four consecutive days of treatment (Dawet and Stephen 2014). However, the ethanol and aqueous extracts of the *P. kotschyi* stem bark exhibited lower activity against the malaria parasite (39.43% and 28.36% reduction in parasitemia, respectively) (Dawet and Yakubu 2014).

The limonoid derivatives **9** and **10** were reported to display significant *in vitro* activity against chloroquine-resistant *Plasmodium falciparum* with IC_50_ values of 1.36 and 1.77 µg/mL, respectively (Hay et al. 2007). The two compounds also displayed significant antiparasitic activity against *Leishmania donovani*, *Trypanosoma brucei rhodesiense* with a low-range IC_50_ of 0.99–3.4 µg/mL. In contrast, the orthoacetatate kotschyin A was found to be inactive against all the tested parasites (Hay et al. 2007). In related work, Sidjui et al. (2018) evaluated the *in vitro* antiplasmodial activity of 14 compounds isolated from *P. kotschyi*. Their findings showed that the limonoid derivatives **9**, **10**, **13**, **14** and **15** exhibited very significant activity against both chloroquine-sensitive (*Pf3D7*) and chloroquine-resistant (*PfINDO*) strains of genus *Plasmodium* with IC_50_ values ranging from 0.75 to 9.05 µg/mL.

Steverding et al. (2020) investigated the trypanocidal and leishmanicidal activities of six limonoids, namely, **9**, **10**, **13**, **14, 15** and **16**, against bloodstream forms of *Trypanosoma bruce*i and promastigotes of *Leishmania major*. All the six compounds showed anti-trypanosomal activity with IC_50_ values ranging from 3.18 to 14.5 µM. Compounds **9**, **10**, **13** and **14** also displayed leishmanicidal activity with IC_50_ of 11.60, 7.63, 2.86 and 14.90 µM, respectively, while **15** and **16** were inactive.

The antiplasmodial, trypanocidal, and leishmanicidal activities of these compounds provide justification for the use of crude extract of *P. kotschyi* in the traditional treatment of malaria and other parasitic infectious diseases.

### Antimicrobial activity

Antimicrobial agents are among the most commonly used medications. The prevalence of antimicrobial resistance in recent years has led to a renewed effort to discover newer antimicrobial agents for the treatment of infectious diseases (Hobson et al. 2021). Extracts of *P. kotschyi* were reported to display appreciable activity against some pathogenic microorganisms.

Ayo et al. (2010) investigated the antimicrobial activity of petroleum ether, ethyl acetate and methanol extracts of the *P. kotschyi* leaves against *Staphylococcus aureus*, *Salmonella typhi*, *Streptococcus pyogenes*, *Candida albicans*, and *Escherichia coli*. The results of the study showed that the ethyl acetate extract exhibited antibacterial activity against all the tested organisms with MIC values of 10–20 mg/mL. In an another similar study, the crude methanol extract of the stem bark of this plant was also shown to exhibit good activity against a panel of pathogenic bacteria and fungi which include methicillin-resistant *S. aureus* (MRSA), *S. aureus*, *S. pyogenes*, *Corynebacterium ulcerans*, *Bacillus subtilis*, *E. coli, S. typhi*, *Shigella dysenteriae*, *Klebsiella pneumoniae*, *Neisseria gonorrhoeae*, *Pseudomonas aeruginosa*, *C. albicans*, *C. krusei*, and *C. tropicalis* with MIC values of 3.75–10.0 mg/mL (Alhassan et al. 2014). The methanol extract of the woody stem was also found to possess antifungal activity against *C. krusei* ATCC 6825 with an MIC of 6.25 mg/mL (Adeniyi et al. 2010).

The secotirucallane triterpenes (compounds **28**, **29** and **30**) isolated from the bark of *P. kotschyi* have been reported to possess significant antibacterial activity against *Staphylococcus aureus* ATCC 25923), *Escherichia coli* S2(1) and *Pseudomonas aeruginosa* with MIC ranging from 6 to 64 µg/mL. Compound **29** exhibited the highest antibacterial activity while **30** had the lowest (Mambou et al. 2018). The presence of these compounds is likely responsible for the antimicrobial property of *P. kotschyi* extracts and justify the ethnomedicinal use of this plant as a chewing stick for tooth cleaning and enhancement of oral health.

### Antioxidant and hepatoprotective activities

The ethanol extract of *P. kotschyi* stem bark has been reported to possess DPPH radical scavenging activity with an IC_50_ of 4 µg/mL (Alain et al. 2014).

A study on the hepatoprotective activity of methanol and aqueous extracts of the *P. kotschyi* leaves revealed that both extracts (at a dose of 750 mg/kg/day) were able to protect the liver against paracetamol induced oxidative damage (Eleha et al. 2016). A similar study conducted by Nchouwet et al. (2018) showed that 2 weeks pre-treatment with aqueous and methanol extracts of *P. kotschyi* stem bark (150 mg/kg/day) significantly suppressed the development of paracetamol induced hepatotoxicity in experimental rats.

### Hypoglycaemic and digestive enzyme inhibitory activities

Diabetes mellitus is disorder associated with abnormal glucose metabolism resulting from insulin insufficiency or dysfunction. It is one of the major non-communicable diseases that affect millions of people globally. Scientific investigation has revealed that *P. kotschyi* extracts possess some antidiabetic properties. Georgewill and Georgewill (2019) investigated the hypoglycaemic effect of aqueous extract of *P. kotschyi* leaves on alloxan induced diabetic rats. Results of their investigation revealed that oral administration of the extract (200 mg/kg/day for 14 days) caused significant hypoglycaemic effect in the experimental animals. The ethanol extract of roots of this plant was also reported to exhibit inhibitory activity against α-glucosidase (IC_50_ = 5.0 ± 0.2 μg/mL), an important digestive enzyme targeted in diabetes treatment (Bothon et al. 2013).

### Antiproliferative activity

Cancer is an important disease that is characterised by the abnormal rapid proliferation of cells that invade and destroy other tissues (Alhassan et al. 2018). It is a major public health problem throughout the world. Pharmacological studies have shown that *P. kotschyi* possesses anticancer potential.

Kassim et al. (2015) investigated the antiproliferative activity and apoptosis induction effect of aqueous extract of *P. kotschyi* roots against a panel of prostate cancer cell lines, namely, PC3, DU-145, LNCaP and CWR-22 cell lines. Results from the 3-[4,5-dimethylthiazol-2yl]-2,5-diphenyltetrazolium bromide (MTT) assay showed that all four cancer cell lines exhibited a dose-dependent decrease in cell proliferation and viability after treatment with the aqueous extract with IC_50_ values ranging from 12 to 42 µg/mL. The results obtained also showed that LNCaP, PC3, DU-145, and CWR-22 cell lines had 42, 35, 33 and 24% induced apoptotic cells, respectively, after treatment with the same extract. The results of both the antiproliferative and apoptosis assay indicated that the LNCaP cells were the most sensitive to the *P. kotschyi* extract.

Heat shock protein 90 (Hsp90) is a molecular chaperone that is involved in the folding, activation and assembly of several proteins including oncoproteins such as HER2, Survivin, EGFR, Akt, Raf-1, mutant p53 (Calderwood et al. 2006; Dal Piaz et al. 2012). Hsp90 is often overexpressed in cancer cells. It has been demonstrated to play a vital role in tumour progression, malignancy and resistance to chemotherapeutic agents (Zhang et al. 2019). Hence, Hsp90 is recently considered as a viable molecular target for development of new anticancer drugs (Gupta et al. 2019). Phytoconstituents of *P. kotschyi* have been shown to possess significant Hsp90 inhibitory activity. Dal Piaz et al. (2012) investigated the Hsp90 binding capability of several compounds using a surface plasmon resonance (SPR) approach. They found that the limonoid orthoacetates (**1–6**) displayed good binding capability to the protein with compound **4** being the most effective. Compound **4** also exhibited significant anti-proliferative activity against three cancer cell lines, namely, PC-3 (human prostate cancer cells), A2780 (human ovarian carcinoma cells), and MCF-7 (human breast adenocarcinoma cells) with IC_50_ values of 62 ± 0.4, 38 ± 0.7 and 25 ± 1.2 µM, respectively. These findings suggest that Hsp90 inhibition is a mechanism of action for anti-proliferative effects of the limonoids orthoacetates from *P. kotschyi*. These findings provide scientific bases for the future development of new anticancer agents from *P. kotschyi* in the form of a standardised herbal preparation or as a pure chemical entity.

### Antidiarrheal activity

Treatment of diarrhoea comes under one of the common ethnomedicinal uses of *P. kotschyi*. To further verify this claim, Essiet et al. (2016) investigated the antidiarrheal property of ethanol extract of *P kotschyi* leaves in Wistar albino rats. Diarrhoea was induced in the animals using castor oil. Results of the investigation revealed that oral administration of the extract (100, 200 and 400 mg/kg) produced significant (*p* < 0.05) dose-dependent inhibition of induced diarrhoea (67–91%). The results also showed that the administered doses of the extract decreased intestinal transit time by 57–66% while intestinal fluid accumulation was decreased by 68–82%. This finding undoubtedly supports the traditional use of this plant in the treatment of diarrhoea.

### Toxicity

There is a general perception that plant-based medicinal products are natural and thus, very safe for human consumption. However, this notion is wrong because several plants have been shown to produce wide range of adverse reactions some of which are capable of causing serious injuries, life-threatening conditions, and even death (Ekor 2014). Hence, it is of paramount importance to investigate the toxicity profile of traditional medicinal plants as well as their phytoconstituents in order to establish their safety. The study of toxicity is an essential component for new drug development process (Hornberg et al. 2014).

Some toxicological studies have been carried out on the extracts of *P. kotschyi*. Nchouwet et al. (2017) investigated the acute and sub-chronic toxicity of *P. kotschyi* stem bark aqueous extract in albino rats. For the acute toxicity study, the LD_50_ was found to be greater than 2000 mg/kg body weight. The sub-chronic administration of the aqueous extract at a dose of 400 mg/kg body weight/day for 28 days, caused significant increase in total protein and HDL-Cholesterol with concomitant decrease in LDL-cholesterol while other biochemical and hematological parameters were found to be within the normal range. However, histological examination revealed the presence of inflammation and necrosis in the kidney and liver tissues of animals treated with 400 mg/kg body weight-/day of extract while tissue samples from animals treated at lower doses remained normal. This implies that the extract may have exhibited some toxic effect on the kidney and liver tissues at 400 mg/kg body weight while it is relatively safe at lower doses.

Kabiru et al. (2015) conducted a sub-chronic toxicity evaluation of a crude methanol extract of leaves in Sprague-Dawley rats at doses of 40, 200 and 1000 mg/kg body weight/day for 4 weeks. They found that the extract did not produce any significant alteration in both hematological and biochemical parameters when compared with standard controls. This implied that the extract was relatively non-toxic at the tested doses. Ezeokpo et al. (2020) also carried out a similar study with an ethanol extract of *P. kotschyi* leaves in Wistar rats. Their results revealed that the extract (400 mg/kg body weight/day) did not produce any significant derangement in hematological and biochemical parameters after 28 days of treatment. The above findings indicated that methanol and ethanol extracts of *P. kotschyi* leaves are relatively non-toxic at higher doses compared to the aqueous stem bark extract. However, more detailed research is still required to corroborate this finding. Albeit most of the pharmacological activities and chemical constituents reported on this plant have been obtained from the leaf’s extracts, the toxicity evaluation of ethanol, methanol and chloroform extracts of roots and stem bark of *P. kotschy* is yet to be carried out and reported. Hence, further toxicity studies on different extracts, fractions and chemical constituents of the root and stem bark of *P. kotschyi* are still required to ascertain the thorough safety of the plant.

## Conclusions

*P. kotschyi* is an important medicinal plant which is used in the traditional treatment of different ailments. Based on its ethnomedicinal claims, extensive pharmacological and phytochemical investigations have been carried out which led to the isolation and characterisation of several bioactive constituents. Results from the pharmacological investigations on this plant and its phytoconstituents have demonstrated its high therapeutic potential in the treatment of cancer and tropical diseases, particularly malaria, leishmaniasis and trypanosomiasis. Although, experimental data support the beneficial medicinal properties of *P. kotschyi*, there were no sufficient data on the toxicity and safety profile of the plant. Nonetheless, this review provides the foundation for future work. Considering the amount of knowledge so far obtained on the medicinal properties of *P. kotschyi*, further studies on this plant should be directed towards establishing its safety profile as well as design and development of drug product either as single chemical entity or as a standardised herbal preparation. Tropical diseases are among the most neglected health problems in the world. The pharmaceutical industries show little research and development interest in this area albeit the devastating effect of such diseases. Therefore, research finding of this nature should be advanced towards the development of useful medicinal products.
